# Preclinical efficacy of the Wnt/β-catenin pathway inhibitor BC2059 for the treatment of desmoid tumors

**DOI:** 10.1371/journal.pone.0276047

**Published:** 2022-10-14

**Authors:** Danielle Almeida Braggio, Fernanda Costas C. de Faria, David Koller, Feng Jin, Abeba Zewdu, Gonzalo Lopez, Kara Batte, Lucia Casadei, Meng Welliver, Stephen K. Horrigan, Ruolan Han, Jeffrey L. Larson, Anne M. Strohecker, Raphael E. Pollock

**Affiliations:** 1 Program in Translational Therapeutics, The James Comprehensive Cancer Center, The Ohio State University, Columbus, OH, United States of America; 2 Department of Surgery, The Ohio State University, Columbus, OH, United States of America; 3 Department of Radiation Oncology, The Ohio State University, Columbus, OH, United States of America; 4 Iterion Therapeutics, INC., Houston, TX, United States of America; 5 Program in Molecular Biology and Cancer Genetics, The James Comprehensive Cancer Center, The Ohio State University, Columbus, OH, United States of America; 6 Department of Cancer Biology and Genetics, College of Medicine, The Ohio State University, Columbus, OH, United States of America; Universite de Nantes, FRANCE

## Abstract

Mutation in the *CTNNB1* gene, leading to a deregulation of the WTN/β-catenin pathway, is a common feature of desmoid tumors (DTs). Many β-catenin inhibitors have recently been tested in clinical studies; however, BC2059 (also referred as Tegavivint), a selective inhibitor of nuclear β-catenin that works through binding TBL-1, is the only one being evaluated in a clinical study, specifically for treatment of desmoid tumor patients. Preclinical studies on BC2059 have shown activity in multiple myeloma, acute myeloid leukemia and osteosarcoma. Our preclinical studies provide data on the efficacy of BC2059 in desmoid cell lines, which could help provide insight regarding antitumor activity of this therapy in desmoid tumor patients. *In vitro* activity of BC2059 was evaluated using desmoid tumor cell lines. *Ex vivo* activity of BC2059 was assessed using an explant tissue culture model. Pharmacological inhibition of the nuclear β-catenin activity using BC2059 markedly inhibited cell viability, migration and invasion of mutated DT cells, but with lower effect on wild-type DTs. The decrease in cell viability of mutated DT cells caused by BC2059 was due to apoptosis. Treatment with BC2059 led to a reduction of β-catenin-associated TBL1 in all mutated DT cells, resulting in a reduction of nuclear β-catenin. mRNA and protein levels of *AXIN2*, a β-catenin target gene, were also found to be downregulated after BC2059 treatment. Taken together, our results demonstrate that nuclear β-catenin inhibition using BC2059 may be a novel therapeutic strategy for desmoid tumor treatment, especially in patients with *CTNNB1* mutation.

## Introduction

Desmoid tumors (DTs) are rare fibroblastic lesions that can occur anywhere in the body. Although considered benign due to their lack of metastatic potential, DTs have a high risk of local recurrence and can be very locally invasive, thereby significantly decreasing patient quality of life. The estimated incidence in the general population is two to four per million population per year [1]. Desmoid tumor patients, predominantly young adults, often experience chronic pain, organ dysfunction and even death [2], and can experience significant psychological and economic effects on their lives. These impacts manifest in chronic use of opioids, social isolation, deleterious changes in education and employment and sleeplessness, anxiety, and depression related to uncertainties in medical management and the unpredictable natural history of the disease [3]. A common feature of desmoid tumors is a dysregulated Wnt/β-catenin pathway mainly caused by gain-of-function mutations in exon 3 of the *CTNNB1* gene (encoding for β‐catenin) which results in nuclear accumulation of β-catenin [4]. Mutations in the β-catenin gene have also been associated with many other more common cancers, e.g., colorectal, melanoma, breast, endometrial, and hepatocellular tumors [5–8], exemplifying both the relevance as well as the urgency of research in this area.

While many therapeutic options are available, the standard treatment for desmoids remains uncertain, due to their rarity, there is a lack of randomized clinical trials to inform therapeutic comparisons [9]. When feasible, resection has historically been the mainstay of treatment for desmoid tumors. A “watchful waiting” approach has been implemented in some centers if the lesion is not causing functional difficulty [10]; however, due to the unpredictable natural history and clinical behavior of this disease, there is controversy in crafting a universal treatment strategy for DT patients [11]. A single desmoid tumor can alternate between phases of rapid growth followed by stabilization or spontaneous regression and then subsequent regrowth [12]. Other modalities relevant to desmoid management include radiation and systemic therapies, such as chemotherapy and targeted therapies [13–17]; however, overall response to most treatment options remains modest, suggesting an ongoing need for better and more individualized approaches.

Giving the importance of Wnt/β-catenin pathway in many malignancies, several drugs to inhibit the β-catenin gene have been developed. However, there are significant challenges in targeting the Wnt/β-catenin pathway, including identifying effective compounds that do not affect β-catenin-mediated normal functions in cellular repair and tissue homeostasis [18–20] Several β-catenin inhibitors are currently being evaluated in clinical trials. BC2059 (an anthracene-9,10-dione dioxime compound: 2-((3R,5S)-3,5dimethylpiperdin-1ylsulfonyl)-7-((3S,5R)-3,5-dimethylpiperidin-1-ylsulfonyl) has emerged as the only nuclear β-catenin inhibitor currently being evaluated in a first in human phase I clinical trial focusing specifically on patients with progressive desmoid tumors [21]. BC2059 is a novel Wnt/β-catenin pathway inhibitor that disrupts β-catenin binding to Transducin β-like protein 1 (TBL1), thereby facilitating β-catenin destruction [22]. Studies have shown that TBL1 is a key player in enhancing the canonical Wnt signaling pathway via direct binding to β-catenin and recruiting it to the promoter of Wnt target genes, resulting in uncontrolled cell proliferation and survival [23, 24]. TBL1 has also been shown to protects β-catenin from proteasomal degradation through binding to a S-phase kinase-associated protein 1 (SKP1)/Cullin-1(CUL1)/F-box protein complex (SCF complex) [25, 26]. In this study, we demonstrate that BC2059 has significant antitumor efficacy via proapoptotic effects as seen in *CTNNB1*-mutated desmoid cell lines. We have also confirmed BC2059-related decreased nuclear β-catenin levels and the decreased expression of Axin2, a downstream target of β-catenin after BC2059 treatment. In addition, BC2059 decreased cell viability and β-catenin activity in an *ex vivo* desmoid tumor model, thus providing possible predictive insights regarding antitumor activity of this therapy in desmoid tumor patients.

## Materials and methods

### Cell strains and reagents

All DT cells included in this study were created in the Sarcoma Research Lab at the Ohio State University and the MD Anderson Cancer Center. This study was conducted with approval from both the Ohio State University and the MD Anderson Cancer Center institutional review board (IRB) with written informed consent of patients. All cell lines and corresponding tumors were genotyped for *CTNNB1* by Sanger sequencing to confirm that the cell lines were truly desmoid tumor cells. Iterion Therapeutics (formerly BetaCat Pharmaceuticals) provided BC2059.

### Cell viability and proliferation assays

CellTiter-Glo Luminescent Cell Viability Assay (Promega) assessed cell viability after six days of exposure to BC2059 for the mutated-DT cells and after thirty days for the *CTNNB1* wild-type cells. The IC_50_ values were determined using GraphPad Prism Version 6.05 software. The capacity to migrate and invade was evaluated as previously reported [27]. Briefly, 8μm trans-well ThinCerts™ migration chambers (Greiner Bio-One) and 8μm Corning Matrigel Invasion Chambers (Corning) were used for evaluating cellular migration and invasion, respectively. Cells were simultaneously seeded and treated with PBS, DMSO, or BC2059 for 24h. The next day, treated cells were plated into the upper migration and invasion chambers in 200 μL plain DMEM. DMEM with 5% FBS (Gemini Bioproducts) was used as the chemoattractant. Endpoint staining was set for 24h for migration assay and 48h for invasion evaluation after seeding, followed by fixing cells in 0.5% crystal violet solution (Fisher Scientific). For quantitative analysis, each well was divided in 4 quadrants and the invaded and migrated cells were counted.

### Flow cytometry and apoptosis analysis

Cell cycle progression and apoptosis induction using Annexin V-PI staining (BD Biosciences) were measured as previously described [28]. Caspase 3/7 apoptosis activity was measured using Incucyte software (Essen Biosciences) as previously described (Braggio, Cancer, 2019). Briefly, apoptotic index was measured by dividing the fluorescence of caspase 3/7 substrate by total number of cells measured using Vybrant® DyeCycle™ Green stain (Life Technologies). Data were analyzed using Incucyte software (Essen Biosciences).

### Immunoprecipitation assays

The collected cells were lysed in 1x lysis buffer (cell signaling) containing protease inhibitor. The immunoprecipitation was performed using the PureProteome™ Magnetic Bead according to the manufacturer’s instructions. Briefly, the protein lysate was incubated with 2μg of the β-catenin antibody (Cell Signaling, D10A8 XP®, cat number #8480) overnight at 4°C. PureProteome™ Magnetic Bead (Millipore) was added to a 1.5 mL microcentrifuge tube and washed with binding buffer using the PureProteome™ Magnetic Stand (Millipore). The antibody-antigen sample was added to the beads, and the mixture was incubated at room temperature for 30 minutes with continuous mixing. Finally, 40 μl of loading buffer was added to the beads and boiled for 10 minutes. Elutions were collected using the PureProteome™ Magnetic Stand and loaded into the SDS-PAGE gel for protein analysis. TBL-1 (Santa Cruz) antibody was used for the immunoprecipitation assay.

### Protein analysis

Western blotting analysis were performed as previously described [29]. Briefly, protein (8-30μg) was separated and transferred to PVDF membranes. Membranes were incubated overnight at 4°C with the indicated antibodies: TBL-1, GAPDH (Santa Cruz), β-catenin and Axin2 (Cell Signaling). For Odyssey CLx imaging, blots were incubated with secondary donkey anti-rabbit or donkey anti-mouse (IRDye 800CW) and donkey anti-goat (IRDye 680RD) (Li-Cor). Nuclear and Cytoplasmic portions were extracted with NE-PER* Nuclear and Cytoplasmic Extraction Kit (Thermo Scientific, Rockford, IL) according to the manufacturer’s instructions. Western blot images presented are representative of 3 independent experiments.

### Quantitative real-time PCR

Total RNA was extracted using RNeasy Mini Kit (Qiagen) following the manufacturer’s instructions. cDNA was generated using TaqMan® Reverse Transcription Reagents (ThermoFisher) and analyzed by quantitative real-time PCR using StepOnePlus™ Real-Time PCR System, using *Axin2* probe (Hs00610344) (ThermoFisher). Relative expression levels were normalized against β-actin and GAPDH RNA expression.

### Explant tissue slice culture

Explant culture was performed as previously described [29]. Briefly, tissue cores were generated with from fresh patient tissue. Tissue cores were cut into slices (400μm) and placed in 96-well plates with 200μL of complete media. Tissue slices were treated with 100 nM of BC2059 and analyzed for cell survival using AlamarBlue® Cell Viability Reagent (Thermo Fisher Scientific). Protein was isolated using Precellys® Evolution homogenizer, Precellys® Lysing Kit (Bertin Instruments).

### Statistical analysis

Unpaired two-tailed Student *t* test analysis assessed statistical significance between experimental groups. For cell viability analysis, Two-way ANOVA test followed by Tukey post-test were performed. *P* < 0.05 was considered statistically significant.

### Ethics approval

This study was conducted with approval from both the Ohio State University and the MD Anderson Cancer Center institutional review boards (IRB).

## Results

### Antitumor efficacy of BC2059 in desmoid tumor cells

**Fig 1:**
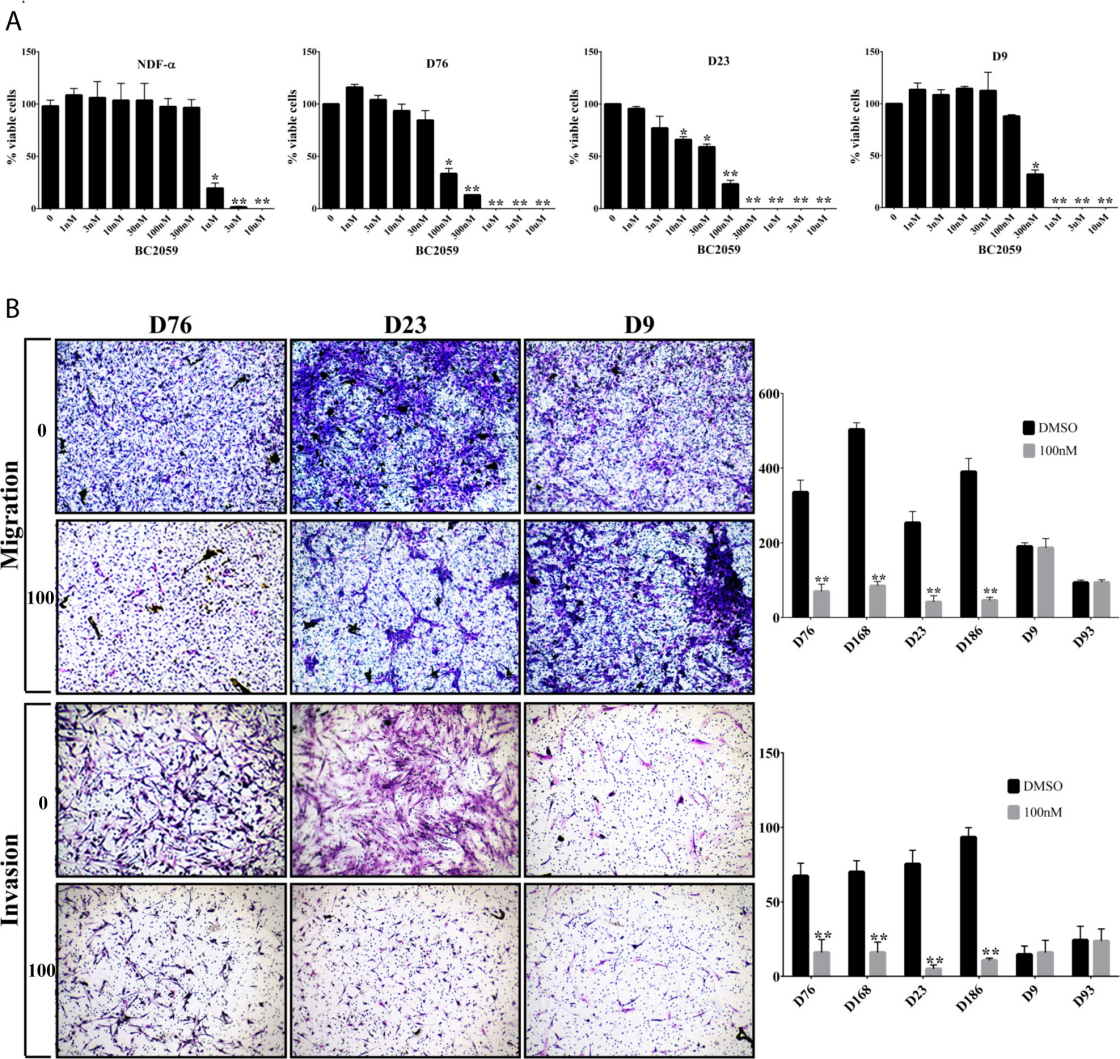
BC2059 efficacy in desmoid cells. A) Desmoid tumor cells strains and normal dermal fibroblasts (NDF-α) were treated with BC2059 (0-10μM) as indicated. B) Representative desmoid tumor migration and invasion in response to BC2059 treatment assessed with Boyden chamber assays. Error bars represent SD from 3 independent experiments. (*P < 0.05; **P < 0.001).

The antitumor efficacy of BC2059 was evaluated in a panel of desmoid tumor cell lines representing the different *CTNNB1* mutations found in desmoid patients (Fig 1A). *CTNNB1* mutated desmoid cells were treated with BC2059 for 6 days. Due to the slow doubling time of wild-type desmoid cells, these cells were treated with BC2059 for 30 days. The IC50 values ranged from 47.79 to 284.7 nM for the desmoid cells and 639.6 to 839.4 nM for the normal cells (HuMSC and NDF-α). BC2059 exhibited potent cytotoxicity, particularly against cell lines bearing *CTNNB1* mutations, with IC50 values in the low nM range (Table 1). The difference in IC50 values comparing cell lines with mutated *CTNNB1* versus those with wild-type *CTNNB1* was statistically significant (*P* < 0.001). We next evaluated the impact of BC2059 on desmoid tumors migration and invasion. After BC2059 treatment, *CTNNB1* wild-type desmoid cells showed markedly less decreases in migration and invasion as compared to mutated DT cells (*P*<0.001) (Fig 1B).

**Table 1:** IC_50_ values of BC2059-treated normal cells lines and desmoid cell strains.

Cell line / strain	IC50 (nM)	β-cat mutation	SD±
D13	47.79	S45F	0.045
D23	58.04	S45F	0.033
D180	61.21	T41A	0.035
D91	86.26	T41A	0.077
D76	93.37	T41A	2635
D186	97.40	S45F	135.2
D14	98.76	S45F	558.5
D168	103.8	T41A	0.095
D93	166.1	WT	0.032
D9	191.5	WT	0.023
D38	191.8	WT	0.074
D55	255.1	WT	0.048
D8	284.7	WT	0.037
NDF-a	639.6	-	0.035
HuMSC	839.4	-	45.30

**Fig 2:**
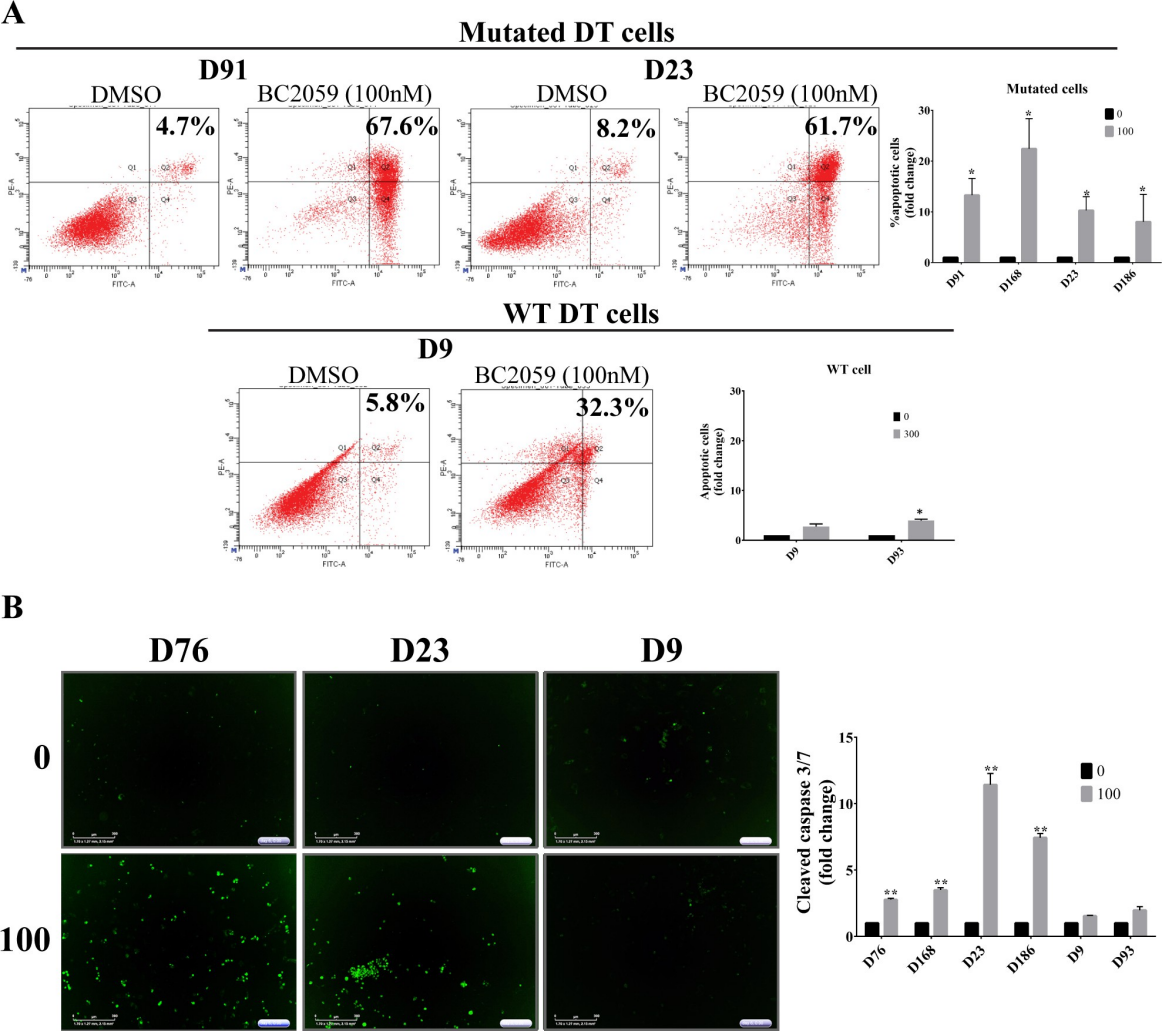
Analysis of BC2059-induced cell death in desmoid cell strains. A) Effects of BC2059 on cell apoptosis were measured by flow cytometry. B) Representative cleaved-caspase 3/7 fluorescent dye images of three desmoid cell strains. Effects of BC2059 on cell caspase-dependent apoptosis were measured using automated IncuCyte imaging. Error bars represent SD from 3 independent experiments. (*P < 0.05; **P < 0.001).

To examine whether BC2059 anti-proliferative effects on DT cells were mediated via cell cycle arrest or by induction of apoptosis, we performed flow cytometric cell cycle and apoptosis analyses. There was no significant changes in cell cycle levels after BC2059 treatment (S1 Fig). Interestingly, apoptosis analysis demonstrated an increase in the percentage of apoptotic cells, but only in the mutated-DT cells (Fig 2A). To confirm this result and to investigate if the observed apoptosis was caspase-dependent, we analyzed caspases-3 and -7 cleavage and observed an increased cleaved caspases-3 and -7 in the *CTNNB1*-mutated subset of DT cells that underwent apoptosis as per the previous flow analysis (Fig 2B). Our results again showed that *CTNNB1* wild-type DT cells did not undergo apoptosis induction after BC2059 treatment, implying that BC2059 treatment might be especially beneficial to desmoid patients harboring a *CTNNB1* mutation.

### BC2059 inhibition of β-catenin binding to TBL-1 decreases β-catenin activity

Recent evidence indicates that BC2059 disrupts the binding of β-catenin to transducin β-like protein 1 (TBL1) (S2 Fig), resulting in reduction of β-catenin levels in the cytoplasm and nucleus [30]. Therefore, we investigated whether BC2059 had a similar effect on desmoid tumor cells. First, we examined the ability of BC2059 to block β-catenin binding to TBL1.

**Fig 3:**
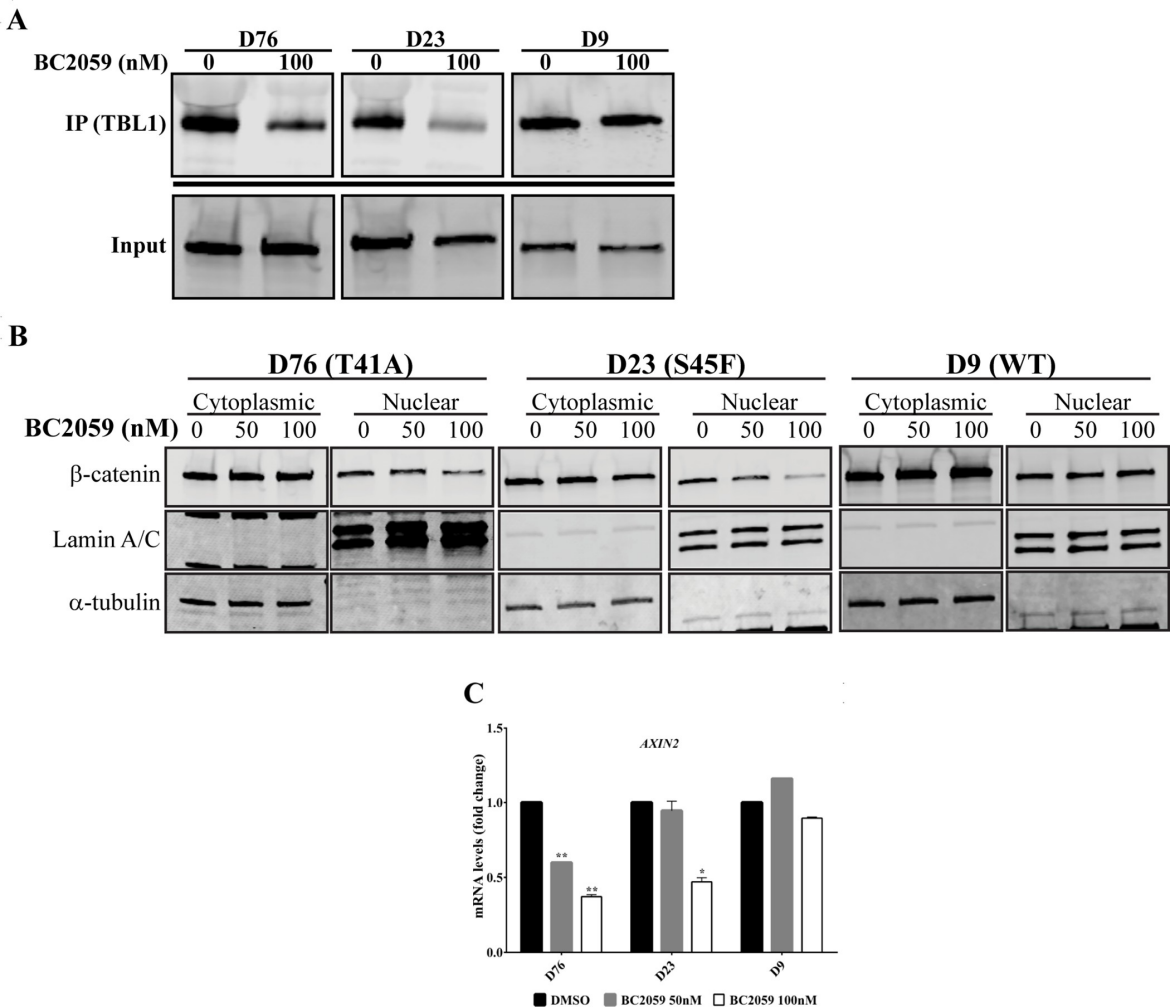
BC2059 inhibits the binding of β-catenin to TBL-1 resulting in decreased β-catenin activity. A) TBL1 expression after co-immunoprecipitation (IP) of β-catenin. Lysates from three desmoid cell lines treated with 100 nM of BC2059 or vehicle were subjected to β-catenin immunoprecipitation by magnetic beads using a specific anti-β-catenin antibody. Whole-cell lysates (input) and immunoprecipitants were analyzed by immunoblotting with an anti-TBL1 antibody. B) Cytoplasmic and nuclear expression of β-catenin in 3 DT cell lines after treatment with BC2059 for 96h. C) mRNA levels of *AXIN2* measured by quantitative real-time PCR after 48h treatment with BC2059. Error bars represent SD from 3 independent experiments. (*P < 0.05; **P < 0.001).

Treatment with BC2059 resulted in decreased whole-cell binding of β-catenin to TBL1 in *CTNNB1*-mutated desmoid cells; however, no significant effect was observed in *CTNNB1* wild-type cells (Fig 3A), probably because desmoid tumor cell lines lacking detectable *CTNNB1* mutation could primarily be composed of fibroblasts cells. To better understand the effects of BC2059 on β-catenin levels, we treated desmoid cells with BC2059 for 96 hours. Western blot analysis showed significantly decreased nuclear β-catenin in *CTNNB1*-mutated cells. Again, no significant effects on nuclear β-catenin were observed in the *CTNNB1* wild-type cell. The *CTNNB1* mutation inhibited the destruction complex, which targets cytoplasmic β-catenin for degradation under unstimulated conditions by phosphorylating β-catenin; as expected, the levels of cytoplasmic β-catenin showed no significant change (Fig 3B). To further investigate whether BC2059 affected the transcriptional activity of β-catenin, desmoid cells were cultured for 48 hours in the presence of BC2059. Quantitative real-time PCR analysis showed a striking reduction in the mRNA levels of the downstream β-catenin transcriptional target Axin2 in the *CTNNB1*-mutated cells but no significant difference in the wild-type cell (Fig 3C).

### BC2059 demonstrates significant antitumor efficacy in an ex vivo model of desmoid tumor

**Fig 4:**
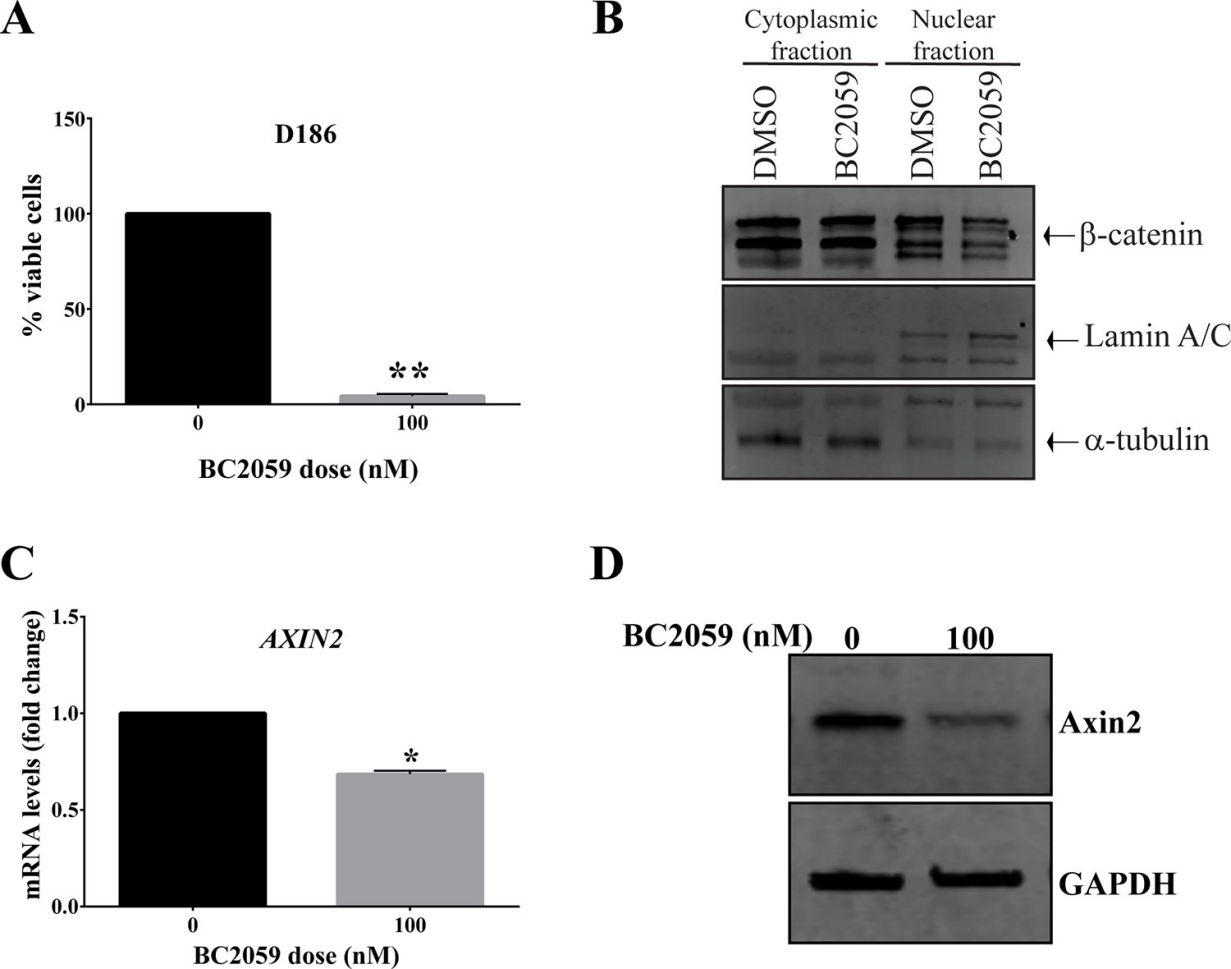
BC2059 efficacy in desmoid explant cell culture. A) Cell survival analysis (alamar blue) of S45F-mutated DT tissue treated *ex vivo* with 100 nM of BC2059 for 96h. B) Representative cytoplasmic and nuclear expression of β-catenin in S45F-mutated DT tissue treated *ex vivo* with 100 nM of BC2059 for 96h. C) mRNA levels of *AXIN2* measured by quantitative real-time PCR in S45F-mutated DT tissue treated *ex vivo* with BC2059 for 48h. D) Representative protein levels of Axin2 in S45F-mutated DT tissue treated *ex vivo* with increasing doses of BC2059 after 96h. Error bars represent SD from 3 independent experiments. (*P < 0.05; **P < 0.001).

A major challenge in studying desmoid tumors in the preclinical setting is due to the lack of desmoid *in vivo* models. To evaluate the activity of BC2059 in a context that might better mirror the clinical situation of desmoid tumors, we established an *ex vivo model* of desmoid tumor harboring a *CTNNB1* mutation S45F. Potent BC2059 antitumor activity was observed. The explant culture treated with 100 nM of BC2059 had a decrease in the percentage of viable cells compared to the cells treated with vehicle alone (Fig 4A). Similar to the results observed in the cell lines, β-catenin nuclear levels were lower in the explant desmoid tissue treated with BC2059 as compared to vehicle only-treated desmoid explant tissue. As before, the levels of cytoplasmic β-catenin had no significant change (Fig 4B). Likewise, the *AXIN2* mRNA levels were significantly lower in the explant-tissue treated with BC2059 as compared to the vehicle-treated tissue (Fig 4C and 4D), suggesting a reduction in β-catenin-specific transcriptional activity. Taken together, our results show that BC2059 might be useful as a future treatment for desmoid tumors patients, especially for those harboring the *CTNNB1* mutation.

## Discussion

Selection of treatment strategies for desmoid tumors can be challenging, especially given the unpredictable natural history and clinical behavior of these tumors. In a recent randomized clinical trial, sorafenib was shown to be effective in slowing desmoid patient disease progression [17]. However, to date there is no commonly accepted standard of care in this disease and the integration of therapeutic alternatives such as active surveillance, surgery, radiation, systemic therapies, chemotherapy and other targeted therapies [9, 13–16, 27, 31–33] remains to be accomplished. Overall response to most treatment options remains modest.

The Wnt/β-catenin pathway plays a role in several cancers by promoting tumor growth, contributing to therapeutic resistance, and by helping create a microenvironment favorable for metastasis [34]. Substantial progress has been made in developing approaches to target this pathway, including small molecule inhibitors, natural compounds, and viral-based and antibody-based inhibitors [35–38]. However, targeting Wnt pathway components can be very challenging, mainly because these entities are often involved in vital normal cell functions, e.g., the interaction of β-catenin with E-cadherin, which is essential for cell adhesion [39]. To circumvent these possible side effects, specific inhibitors have been explored that target β-catenin transcriptional activity. In this context, the efficacy of BC2059, a potent inhibitor of nuclear β-catenin activity, has been investigated in tumors with a deregulated Wnt/β-catenin pathway. BC2059 has been shown to inhibit β-catenin activation of several cancer genes, such as *AXIN2* and *BIRC5*, with minimal effects on the maintenance of normal tissue functions [22, 30].

A dysregulated Wnt/β-catenin signaling pathway is a common feature of desmoid tumors [40], and is mainly caused by mutations in the *CTNNB1* gene, resulting in increased β-catenin activity. Targeted therapies focusing on inhibiting the canonical Wnt/β-catenin pathway may provide therapeutic benefit for desmoid tumor patients; however, to date, no β-catenin targeted therapy has been approved for treatment of this disease. However, to the best of our knowledge, the preclinical effect of BC2059 in desmoid tumors has never been published, hence the relevance of these desmoid tumor cell and explant tissue culture studies.

Our data establishes a significant BC2059 dose-dependent inhibition of viability, migration and invasion in a subset of desmoid tumor cells, with IC50 values ranging from 47.79 to 284.7 nM. Preclinical studies predict these doses to be readily achievable drug level in patients. Interestingly, BC2059 was not able to inhibit cell viability of wild-type *CTNNB1*desmoid tumors. This is in contrast to previous studies that showed efficacy of BC2059 in multiple myeloma and acute myeloid leukemia, tumors that typically have a upregulated Wnt/β-catenin pathway, but are not mutated for the *CTNNB1* gene [22, 30].

Comparison of *CTNNB1* mutation between the original tumor and the associated derived cell line was done using Sanger sequencing as the primary method; this helped to also differentiate desmoid tumor cells from fibroblasts. All primary tumors with no detectable *CTNNB1* mutation were identified as wild-type desmoid tumors. Desmoid tumor cell lines lacking detectable *CTNNB1* mutation could primarily be composed of fibroblasts cells and not tumor cells as a possible explanation for the lack of BC2059 effects on DT wild type cell lines. Similar to previous studies, treatment with BC2059 inhibited β-catenin binding to TBL1, resulting in decreased nuclear β-catenin protein levels and attenuated transcriptional activity [22, 30]. However, in contrast to the results shown by Savvidou et al., cytoplasmic levels of β-catenin in desmoid tumors were not been affected by BC2059 [30]. The fact that most desmoid tumors harbor a *CTNNB1* mutation leading to inhibition of β-catenin degradation is possibly an explanation for these contrasting results. Pertinently, BC2059 showed inhibition of cell viability in human mesenchymal stem cells or normal dermal fibroblast cells but only at much higher doses; these latter cell types are negative for active nuclear β-catenin compared to desmoid tumors. In turn, this might suggest the possibility to use minimal, yet effective doses of this drug in the clinic after accounting for any possible off-target effects. BC2059 treatment also selectively induced apoptosis in desmoid tumor cells, further suggesting that this drug might be useful therapeutically in desmoid tumors. To the best of our knowledge, this is the first preclinical study demonstrating the efficacy of BC2059 treatment in both *in vitro* contexts and *ex vivo* desmoid tumors models.

In conclusion, therapy with BC2059 showed a significant efficacy against *CTNNB1*-mutated desmoid cells by inhibiting proliferation, migration, and invasion. The mechanism of BC2059-mediated cell death appears to be through induction of apoptosis. Moreover, desmoid tumors with no detectable mutations of the *CTNNB1* gene are more tolerant to BC2059 as compared to *CTNNB1*-mutated desmoid tumors. Taken together, these findings support further investigations of BC2059 as a possible novel therapeutic approach for desmoid tumor patients.

## Supporting information

S1 Fig Cell cycle distribution of desmoid cells treated with vehicle (DMSO) or 100nM BC2059 for 48h were assessed by flow cytometry (PI staining). (TIF)

S2 Fig BC2059 mechanism of action. Transducin β-like protein 1 (TBL1) recruits β-catenin to the Wnt target gene promoter. BC2059 disrupts the binding of β-catenin to TBL1, facilitating β-catenin destruction, resulting in the inhibition of the Wnt/β-catenin pathway.
(TIF)

S1 Raw images (ZIP)
