# Supplementary figures and images for: Preclinical efficacy of the Wnt/β-catenin pathway inhibitor BC2059 for the treatment of desmoid tumors
Source: PLoS One. 2022 Oct 14;17(10):e0276047. doi: 10.1371/journal.pone.0276047 (PMC9565452; doi:10.1371/journal.pone.0276047)

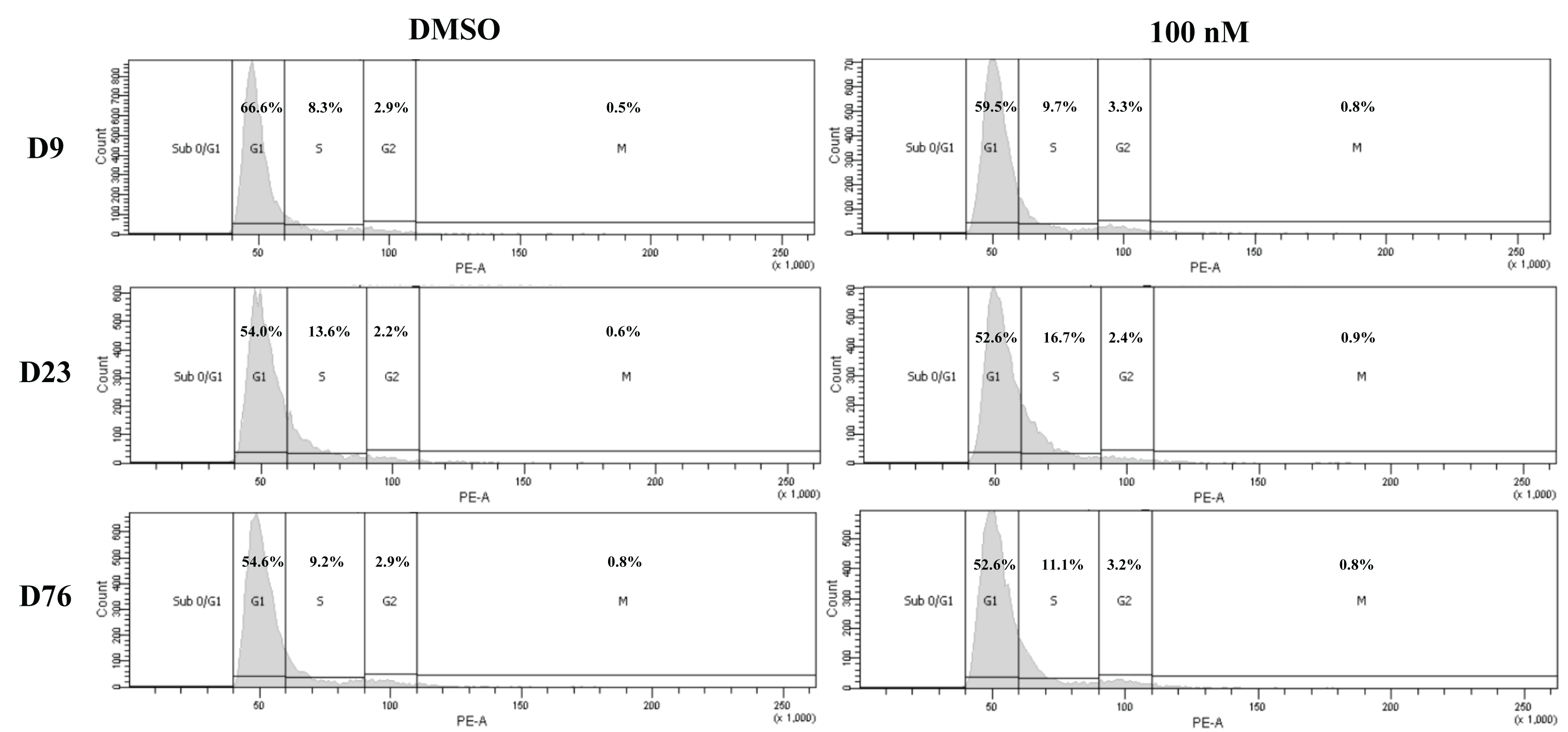

Supplement: S1 Fig — (TIF) [file pone.0276047.s001.tif]

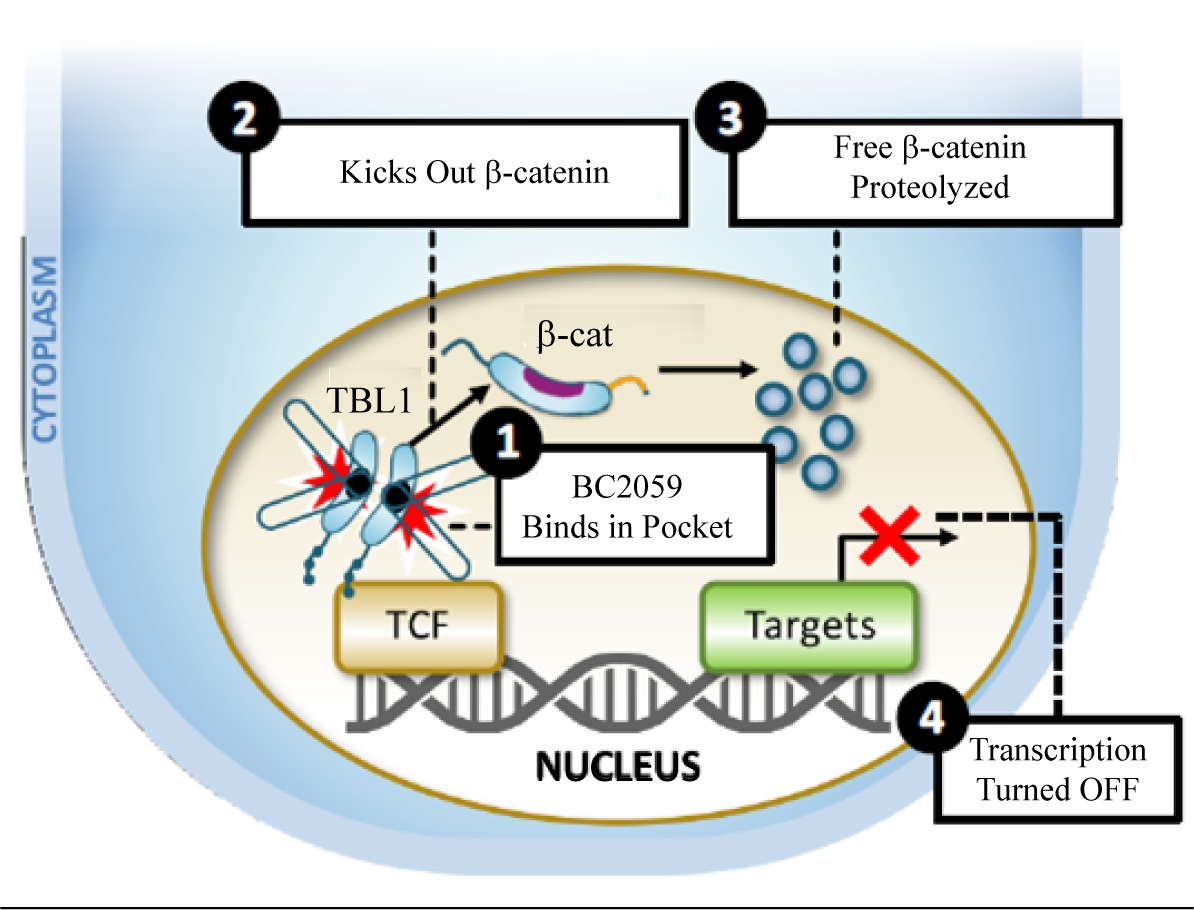

Supplement: S2 Fig — Transducin β-like protein 1 (TBL1) recruits β-catenin to the Wnt target gene promoter. BC2059 disrupts the binding of β-catenin to TBL1, facilitating β-catenin destruction, resulting in the inhibition of the Wnt/β-catenin pathway. (TIF) [file pone.0276047.s002.tif]

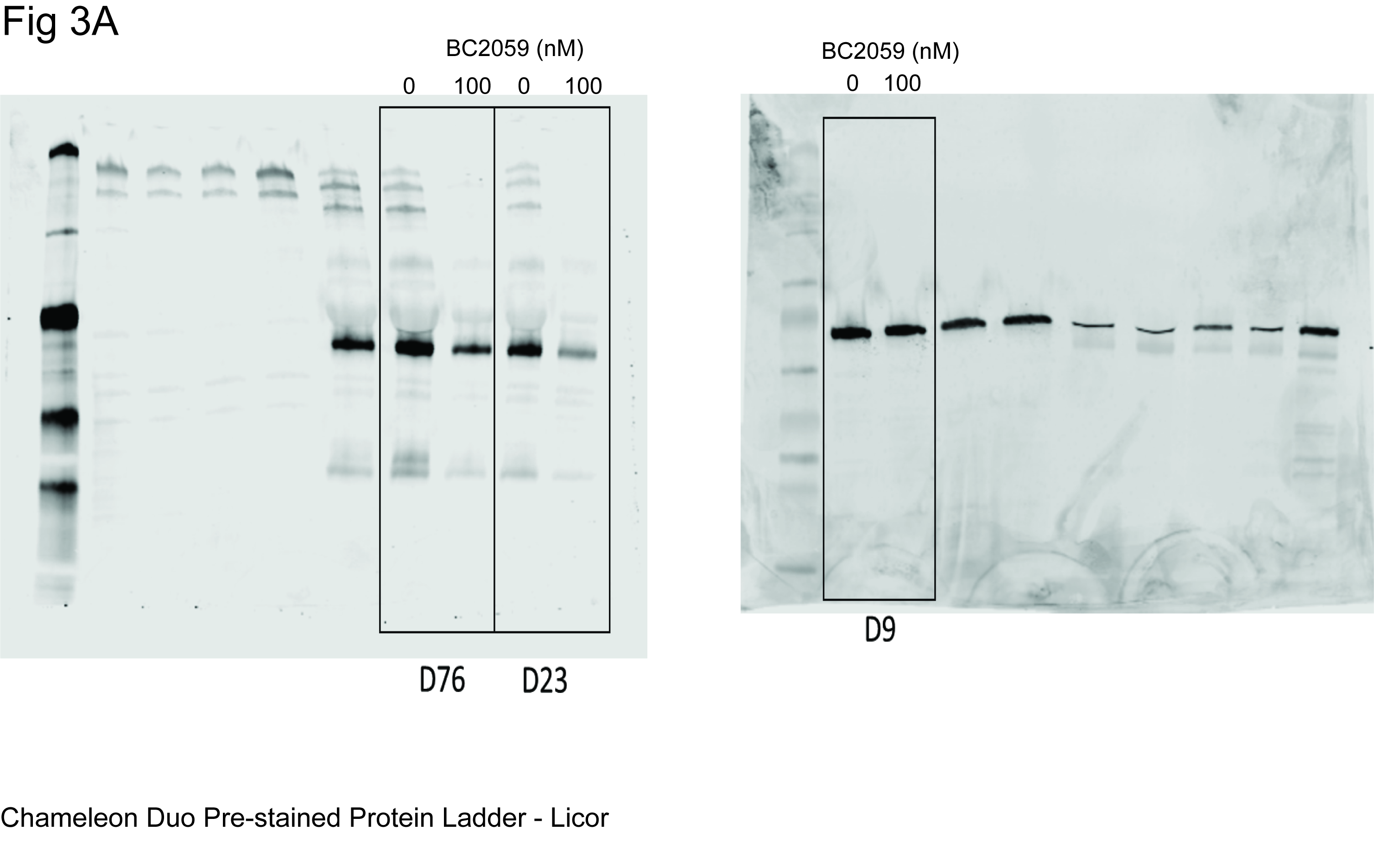

Supplement: S1 Raw images — (ZIP) [file pone.0276047.s003.zip › Uncropped blottings_Braggio et al/Uncropped blotting_Fig 3A.tif]

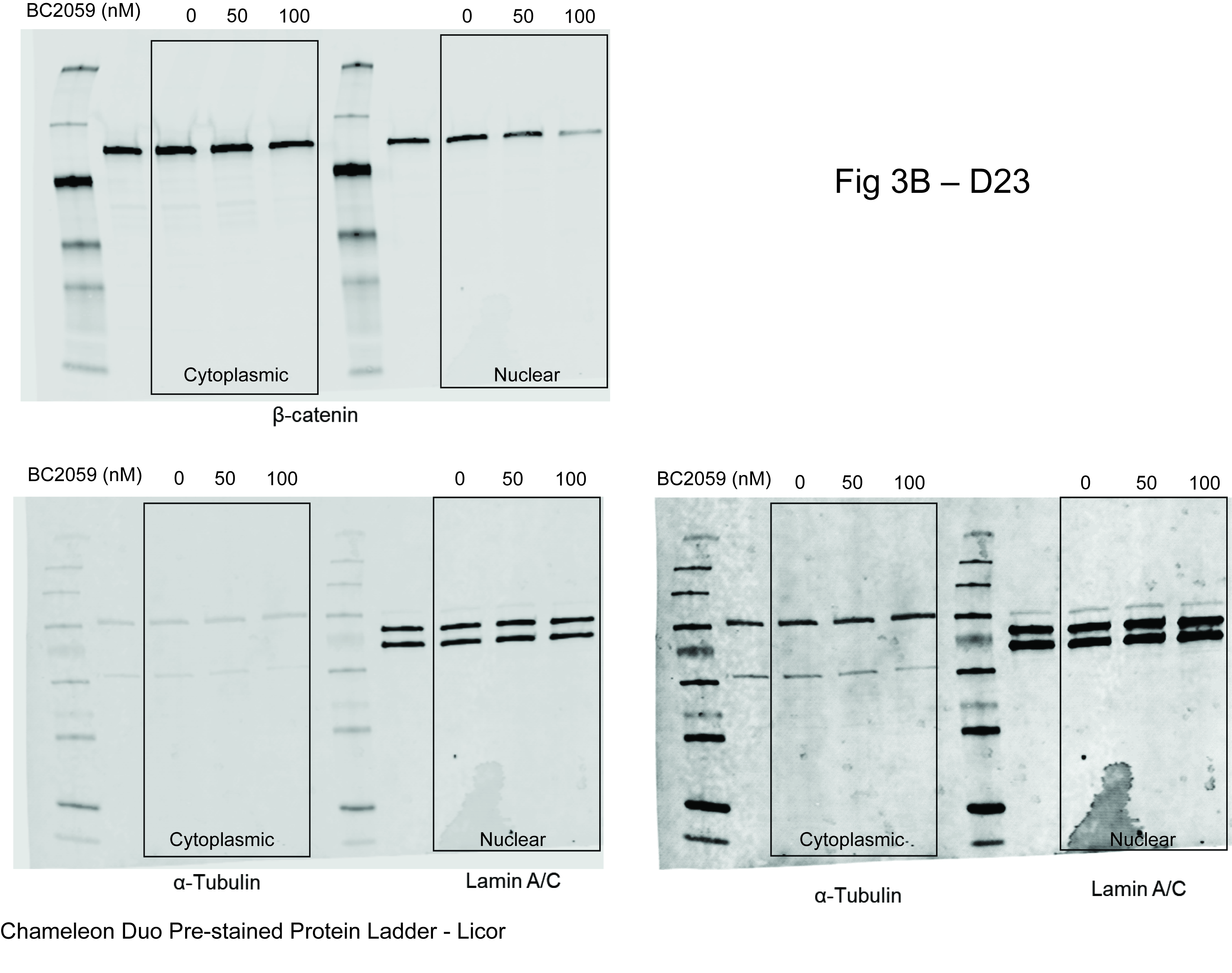

Supplement: S1 Raw images — (ZIP) [file pone.0276047.s003.zip › Uncropped blottings_Braggio et al/Uncropped blotting_Fig 3B_D23.tif]

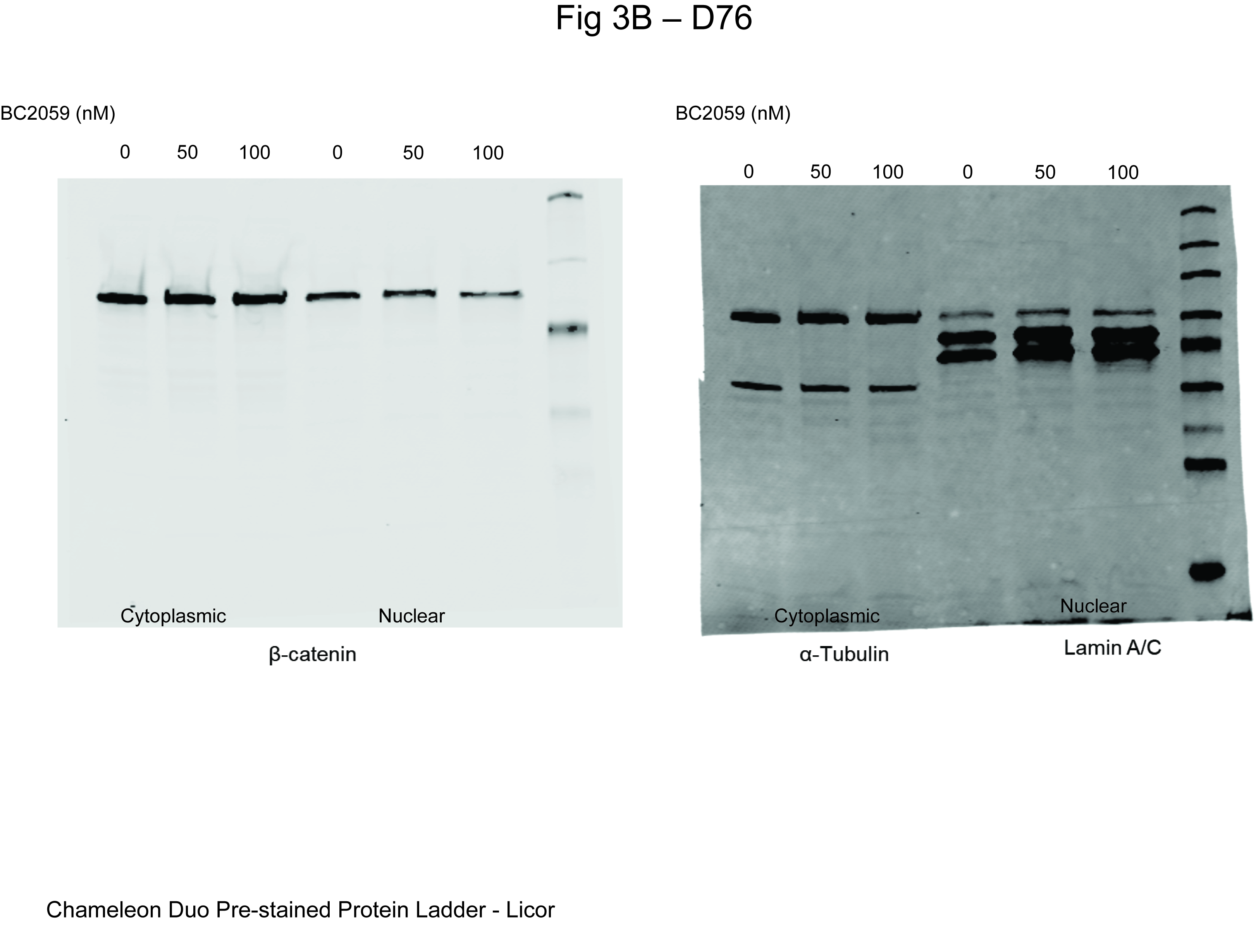

Supplement: S1 Raw images — (ZIP) [file pone.0276047.s003.zip › Uncropped blottings_Braggio et al/Uncropped blotting_Fig 3B_D76.tif]

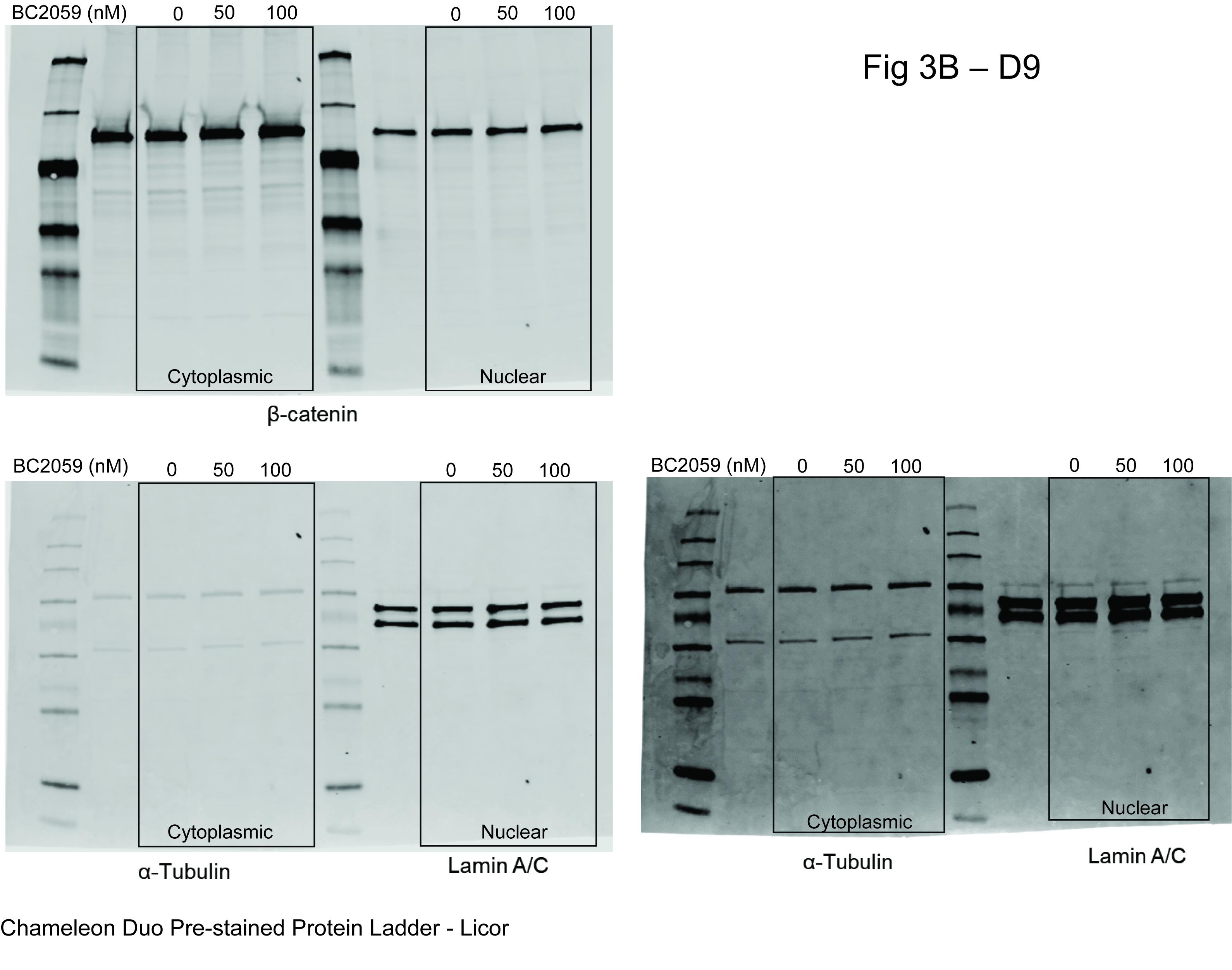

Supplement: S1 Raw images — (ZIP) [file pone.0276047.s003.zip › Uncropped blottings_Braggio et al/Uncropped blotting_Fig 3B_D9.tif]

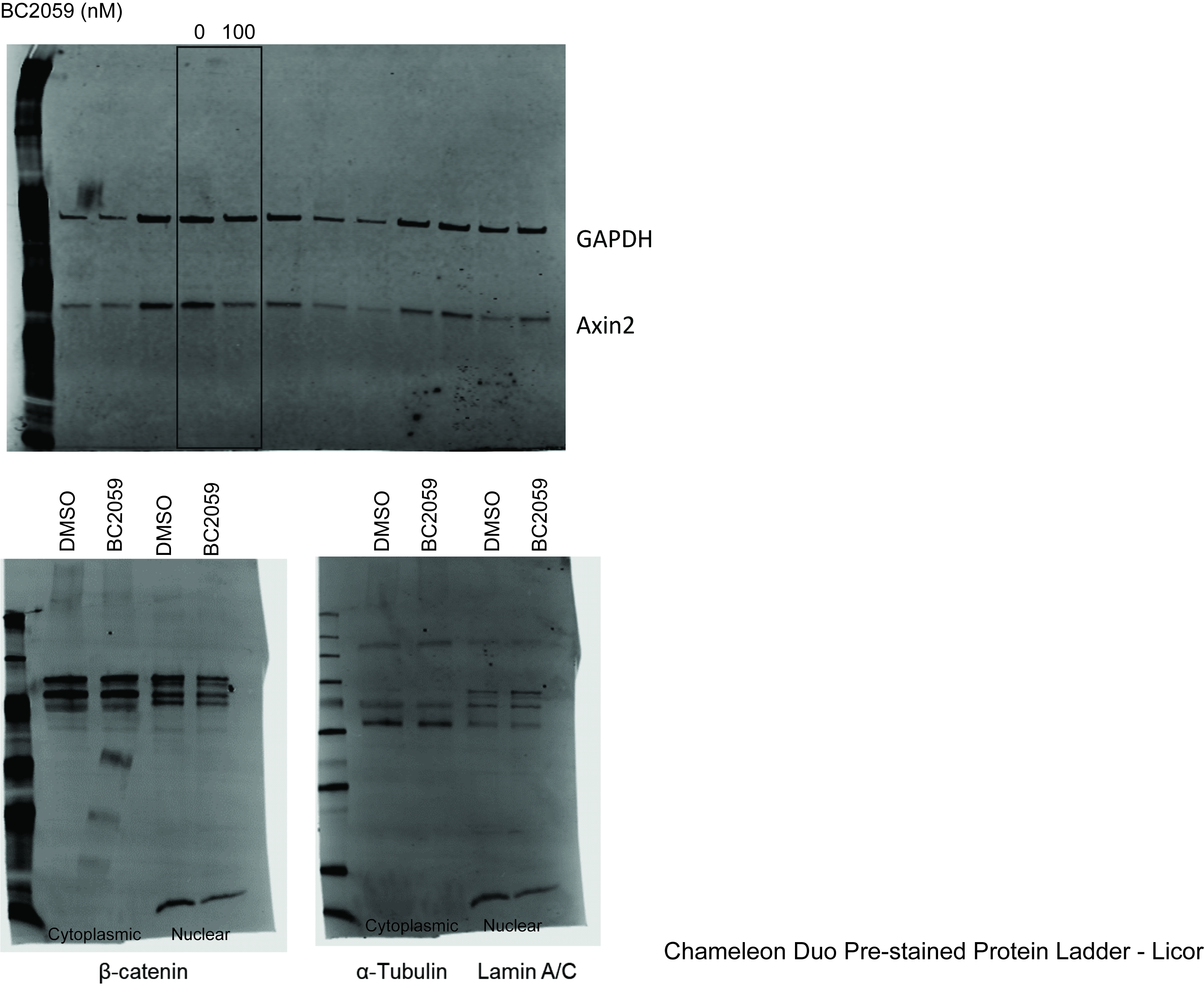

Supplement: S1 Raw images — (ZIP) [file pone.0276047.s003.zip › Uncropped blottings_Braggio et al/Uncropped blotting_Fig 4B and 4D.tif]
